# Okinawa-Based Nordic Diet Decreases Plasma Glial Fibrillary Acidic Protein Levels in Type 2 Diabetes Patients

**DOI:** 10.3390/nu16172847

**Published:** 2024-08-26

**Authors:** Dovilė Pocevičiūtė, Malin Wennström, Bodil Ohlsson

**Affiliations:** 1 Cognitive Disorder Research Unit, Department of Clinical Sciences Malmö, Lund University, 214 28 Malmö, Sweden; dovile.poceviciute@med.lu.se; 2 Department of Internal Medicine, Lund University, Skåne University Hospital, 214 28 Malmö, Sweden; bodil.ohlsson@med.lu.se

**Keywords:** astrocyte, cytokine, neuroinflammation, plasma, gut–brain axis

## Abstract

Elevated levels of glial fibrillary acidic protein (GFAP) in plasma reflect neuroinflammation and are linked to cognitive decline. Preclinical studies show that dietary change can attenuate astrocyte reactivity and neuroinflammation. In the current study, we investigate if the Okinawa-based Nordic (O-BN) diet alters plasma GFAP levels in patients with Type 2 Diabetes (T2D), a metabolic disorder associated with cognitive disturbances and an increased risk of dementia. Plasma GFAP levels were measured in T2D patients (n = 30) at baseline, after 3 months of the diet, and after a subsequent 4 months of unrestricted diets. The GFAP levels decreased significantly after 3 months of the diet (*p* = 0.048) but reverted to baseline levels after 4 months of unrestricted diets. At baseline, the GFAP levels correlated significantly with levels of the neurodegeneration marker neurofilament light polypeptide (r = 0.400*) and, after correcting for age, sex, and body mass index, with proinflammatory plasma cytokines (ranging from r = 0.440* to r = 0.530**) and the metabolic hormone islet amyloid polypeptide (r = 0.478*). We found no correlation with psychological well-being. These results suggest that the O-BN diet reduces neuroinflammation in T2D patients and may thus be an important preventive measure for managing T2D and reducing the risk of neurodegenerative disorders.

## 1. Introduction

Neuroinflammation is an inflammatory response in the brain seen in association with severe damage like traumatic head injuries [1] or neurodegenerative disorders [2] but also in response to milder or more subtle changes, such as major depression, stress, or inflammatory illness [3]. The event is characterized by an activation of microglia and astrocytes, where the former cell type represents the resident innate brain immunity cell [4] and the latter supports neurons in terms of nourishment and environmental homeostasis [5]. Both glial cell types respond to damage by secreting anti- and proinflammatory cytokines, including interleukins (IL) 1β, IL-6, and IL-8, tumor necrosis factor (TNF) α, and interferon (IFN) γ [6]. They also both undergo morphological changes, visualized by swollen cell bodies and retracted processes [4,7,8,9]. These changes are mediated by alterations in proteins that form the cytoskeleton of the glial cells [10,11]. In astrocytes, the glial fibrillary acidic protein (GFAP) is the most well-known and well-studied cytoskeleton protein associated with activation-induced morphological changes. The protein is a type III intermediate filament implicated in a number of essential astrocytic events, including motility, proliferation, vesicle trafficking, interactions with other cells, and maintenance of the blood–brain barrier (BBB) [11]. In response to brain damage and injury, but also progressively during ageing [12,13], the expression of the astrocytic GFAP increases in the brain. While the majority of the increased GFAP is involved in the restructuring of the cytoskeleton, some of the GFAP is cleaved and released extracellularly as fragments [14,15]. The function of these fragments is less known, but it has been suggested that they play a role in modulating neuroinflammatory responses. The GFAP fragments are suggested to be transported by the glymphatic system across the BBB into the blood stream [16], and when the BBB is disrupted, as in the case of an injury, the outflux of GFAP fragments increases [17]. Such an increase in injury-related GFAP fragments has been found in plasma shortly after a traumatic brain injury [17,18] but also in plasma of patients with the neurodegenerative disorder Alzheimer’s disease (AD) [19,20]. The GFAP plasma levels in the latter patient group correlated with disease severity and cognitive decline [21]. Since patients with neurocognitive disorders (poor performance on cognitive tests) also demonstrate enhanced levels of GFAP in their serum [19], GFAP has been proposed as a blood-based biomarker reflecting neuroinflammatory events affecting cognition.

Increased levels of autoantibodies against GFAP have further been found in plasma from patients with Type 2 Diabetes (T2D) [22]. T2D is a chronic metabolic disorder characterized by persistent hyperglycemia and insulin resistance, which in turn cause pancreatic β-cell failure and micro-/macro-vascular complications [23]. Many T2D patients also suffer from cognitive changes, such as memory disturbances [24] and depression [25], and the risk of developing AD later in life is nearly 2-fold [26]. Whether the increase in GFAP autoantibody levels in T2D patients reflects elevated plasma GFAP levels or astrocyte activation in the brain is not determined yet, but preclinical studies have shown increased brain GFAP expression in a wide range of different rodent diabetes models [27]. It is thus tempting to speculate that the increase in GFAP autoantibody levels in T2D patients is caused by elevated levels of brain-derived GFAP, reflecting neuroinflammatory events which may in turn contribute to the cognitive disturbances seen in these patients.

One of the most effective ways to manage T2D is to follow specific diets, in particular diets that are rich in whole grains, vegetables, fruits, nuts, and legumes; moderate in alcohol consumption; and lower in red/processed meats, refined grains, and sugar-sweetened beverages [28]. These diets often result in weight loss, which is associated with beneficial outcomes, such as a reduction in cardiovascular risk and the risk of all-cause mortality. To exemplify, when mice after 6 months of a high-fat diet (a model for T2D/obesity) received a standard diet for 4 months, they lost significant amounts of weight and functionally recovered after stroke [29]. Interestingly, the recovery was associated with decreased diabetes-induced neuroinflammation and reduced astrocyte reactivity [29]. We have previously investigated the impact of the Okinawa-based Nordic (O-BN) diet on T2D, a diet which emphasizes eating whole-grains; non-processed, plant-based foods with a high intake of fiber, fat, and protein; and moderately low carbohydrate energy content [30]. The content of high-glycemic-index food and gluten is reduced in the O-BN diet, which was shown to improve insulin resistance, alter the microbiota composition, and decrease metabolic syndrome [31]. The diet is also rich in omega-3 fats, which were demonstrated to lower total cholesterol, HbA1c, and CRP levels in T2D patients [32]. In our previous studies, the O-BN diet increased satiety and improved gastrointestinal (GI) symptoms, as well as reduced the body mass index (BMI) and levels of metabolic (e.g., glucose, islet amyloid polypeptide (IAPP)) and inflammatory (e.g., IL-18) markers in T2D patients following the diet for 3 months [30,33]. Importantly, the diet also substantially improved the psychological well-being [30]. Since cognitive changes and depression are associated with neuroinflammatory processes and altered levels of plasma GFAP [34,35], we hypothesized that the effect of the O-BN diet on psychological well-being could partly be due to an impact of astrocytic reactivity. Therefore, in the current study, we measured plasma GFAP levels in T2D patients following the O-BN diet for 3 months and analyzed possible correlations with psychological well-being and the neurodegeneration marker neurofilament light polypeptide (NfL) in addition to peripheral metabolic (e.g., glucose, insulin) and inflammation (e.g., cytokines) markers, as well as markers for gut barrier leakage.

## 2. Materials and Methods

### 2.1. Patients Included in the Study

This study was performed on fasting plasma samples from a patient cohort already described in previous studies [30,36,37,38,39,40,41]. The cohort consists of n = 30 T2D patients (n = 17 women) from southern Sweden. The inclusion and exclusion criteria have been described before [36]; in short, patients had parents who were both born in Scandinavia and were included independently of BMI or anti-diabetes treatment regimen. Also, patients with severe food allergies, Type 1 Diabetes, a prior major GI surgery, and severe heart, pulmonary, cardiovascular, malignant, or psychiatric diseases were excluded from the study. The patients were delivered the same diet for 3 months with an exception for breakfast, which they had to prepare themselves. The meal content has previously been described in detail [30]. The plasma samples were collected from n = 30 patients at baseline (0 month), n = 30 patients after 3 months of the diet (3rd month), and n = 23 patients after another 4 months of unrestricted diets (7th month) (further termed as the clinical follow-up). At baseline, n = 4 patients were on anti-depressants, of whom n = 3 had left the study at the clinical follow-up. The *APOE* genotype has been identified and described before [33], and the patients were stratified based on their *APOE4* status. The *APOE4* carriers consisted of *ε34* (n = 8) and *ε24* (n = 3) carriers and non-carriers of *ε33* (n = 14) and *ε23* (n = 5) carriers.

### 2.2. Clinical Characteristics

The mean age of the cohort is 58 ± 8 years, mean disease duration 10 ± 8 years, and mean BMI 29.8 ± 4.2 kg/m^2^ at baseline [30]. Nineteen patients had at least one diabetic complication (retinopathy, microalbuminuria, peripheral neuropathy, macroangiopathy, autonomous neuropathy, or GI dysmotility), of which retinopathy was the most common [36].

### 2.3. Analysis of Variables

The plasma GFAP levels were measured using a commercially available Human GFAP ELISA kit (#MBS2506721; CliniSciences, Copenhagen, Denmark) according to the manufacturer’s instructions. The plasma levels of NfL and metabolic (C-peptide, cholesterol, ghrelin, glucagon, glucagon-like peptide-1, glucose, glucose-dependent insulinotropic polypeptide, HbA1c, high-density lipoprotein, insulin, insulin resistance, islet amyloid polypeptide, leptin, low-density lipoprotein, short fatty acids (acetic, butyric, isobutyric, isovaleric, and propionic), and triglycerides) and peripheral inflammation markers (γ glutamyl transferase, alanine transaminase, albumin, C-reactive protein, calprotectin, cholecystokinin, cytokines (interferon γ, interleukin (IL)1α, IL1β, IL12p70, IL18, IL2, IL4, tumor necrosis factor α), haptoglobin, leukocyte number, plasminogen activator inhibitor-1, resistin, total IgA, visfatin, and zonulin), as well as patients’ psychological well-being, have been evaluated and described in previous studies [30,33,38].

### 2.4. Statistical Analyses

All statistical analyses were performed using the SPSS software (version 29.0.2.0). The plasma GFAP levels were analyzed with either related-samples Wilcoxon Signed Rank Test or Mann–Whitney U Test. The correlations were performed with Spearman and Partial Correlation tests, in the latter adjusting for age, sex, and BMI or age alone. The differences and correlations were considered significant at *p* ≤ 0.05. The power analysis has been described in a previous study [36]. In short, at most n = 9 subjects were needed to show clinically important differences in weight, HbA1c, total cholesterol, and systolic blood pressure (BP) and n = 18 subjects in diastolic BP with 80% power at 5% significance level. In total, n = 30 subjects were included to take any dropouts into account.

## 3. Results

### 3.1. The Okinawa-Based Nordic Diet Decreases Plasma GFAP Levels

**Figure 1:**
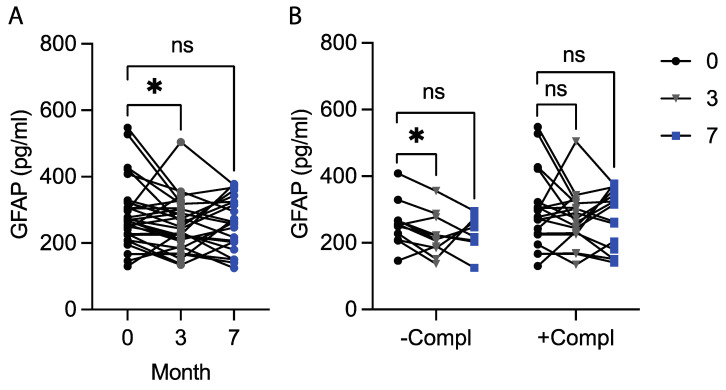
Plasma GFAP levels. The graph illustrates significantly reduced plasma GFAP levels after 3 months of the diet compared to baseline, but the levels at the clinical follow-up are similar to baseline levels (**A**). Stratification of patients with (+compl) and without (−compl) at least one diabetic complication reveals a significant reduction in plasma GFAP levels after 3 months exclusively in the −compl group (**B**). The data are presented as individual values and are analyzed with the related-samples Wilcoxon Signed Rank Test. ns—not statistically significant. *—*p* ≤ 0.05.

We initiated the study by measuring the plasma GFAP levels in T2D patients at baseline, after 3 months of the diet, and after another 4 months of unrestricted diets (the clinical follow-up). The related-samples (Wilcoxon) test revealed significantly decreased GFAP levels after 3 months (*p* = 0.048), but the baseline levels did not differ significantly from the GFAP levels at the clinical follow-up (*p* = 0.370) (Figure 1A). The GFAP levels did not differ between males and females or *APOE4* carriers and non-carriers at baseline, after 3 months, or at the clinical follow-up (Table 1). Interestingly, patients with at least one diabetic complication (+compl) showed significantly higher levels of GFAP after 3 months compared to patients with no diabetic complications (−compl), while this significance in difference was not reached when analyzing the baseline or clinical follow-up values (Table 1). Further analysis using the related-samples test showed that only the −compl group but not the +compl group demonstrated lowered GFAP levels after 3 months compared to baseline (*p* = 0.021 vs. *p* = 0.463) (Figure 1B). However, no difference in GFAP levels at the clinical follow-up compared to baseline in either −compl or +compl groups (*p* = 0.293 vs. *p* = 0.776) was found (Figure 1B).

### 3.2. Plasma GFAP Levels at Baseline Correlate Positively with Plasma NfL Levels but Not with Psychological Well-Being

The plasma GFAP levels correlated positively with NfL at baseline (Table 2) and were close to correlating significantly after 3 months of the diet (r = 0.355, *p* = 0.054), while the correlation was lost at the clinical follow-up (r = 0.258, *p* = 0.235). Since the GFAP levels also correlated with age at baseline (Table 2) and this correlation has been described in previous studies [42,43], we also analyzed the correlations after adjusting for age and age in addition to sex and BMI. The correlation with NfL remained significant after correcting for age and age, sex, and BMI at baseline (Table 2) but not after 3 months (r = 0.224, *p* = 0.243; r = 0.162, *p* = 0.439) or at the clinical follow-up (r = 0.009, *p* = 0.969; r = −0.042, *p* = 0.861). No correlations between GFAP and psychological well-being were found at any of the time points (baseline: r = −0.110, *p* = 0.570, after 3 months: r = −0.010, *p* = 0.959, the clinical follow-up: r = −0.301, *p* = 0.162), regardless of corrections for age (baseline: r = −0.154, *p* = 0.435, after 3 months: r = −0.127, *p* = 0.510, the clinical follow-up: r = −0.277, *p* = 0.213) or age, sex, and BMI (baseline: r = −0.228, *p* = 0.262, after 3 months: r = −0.253, *p* = 0.223, the clinical follow-up: r = −0.288, *p* = 0.218). In addition, the comparative analysis showed no significant difference in plasma GFAP levels between individuals with low scores of psychological well-being (<15, indicative of good well-being) and high scores (≥15, indicative of poor well-being) either at baseline (267.1 (216.7, 415.4) vs. 262.5 (211.9, 302.0), *p* = 0.374), after 3 months of the diet (246.3 (219.4, 306.5) vs. 233.3 (140.7, 322.6), *p* = 0.504), or at the clinical follow-up (273.8 (205.0, 334.0) vs. 211.3 (151.0, 289.7), *p* = 0.319).

### 3.3. Plasma GFAP Levels Correlate Positively with Plasma Levels of Metabolic and Peripheral Inflammation Markers but Not with Gut Leakage Markers 

The GFAP plasma levels also correlated with C-peptide and triglycerides at baseline (Table 2). After age and/or age, sex, and BMI adjustments, we found additional significant positive correlations with IAPP and several cytokines and a negative correlation with HDL (Table 2). No significant correlations were found between GFAP levels and BMI (r = −0.045, *p* = 0.813) or gut leakage markers, i.e., feces albumin/plasma albumin (r = 0.154, *p* = 0.416) or feces zonulin/plasma zonulin (r = 0.287, *p* = 0.124) at baseline or after age (r = −0.058, *p* = 0.767; r = 0.244, *p* = 0.201; r = 0.000, *p* = 1.000, respectively) or age, sex, and BMI (r = na, *p* = na; r = 0.282, *p* = 0.155; r = −0.053, *p* = 0.792, respectively) adjustments. Notably, we found several significant (or close to significant) positive correlations between GFAP and insulin, as well as insulin resistance, after 3 months (r = 0.429, *p* = 0.018; r = 0.370, *p* = 0.044) and at the clinical follow-up (r = 0.408, *p* = 0.053; r = 0.525, *p* = 0.010) before age adjustments.

## 4. Discussion

Our study shows that the plasma levels of GFAP are reduced in T2D patients who follow the O-BN diet for 3 months. This reduction appeared to be the most prominent in patients without diabetic complications, and at the clinical follow-up (when the diet was no longer followed), the GFAP levels returned to baseline levels. This study also shows that the plasma GFAP levels at baseline are associated with the plasma levels of NfL, a biomarker for neurodegeneration, and after adjusting for age, the levels also correlate with the levels of plasma cytokines. No correlation was, however, found with psychological well-being or gut leakage markers. 

The reduction in plasma levels of GFAP in T2D after 3 months of the diet is interesting, as it suggests that a diet can alter the plasma levels of a protein associated with neuroinflammation and cognitive alterations [44]. The hypothesis that a diet affects GFAP expression was introduced already two decades ago, when a study showed that food restriction attenuates age-related GFAP expression in the rat brain [13], but our study is the first to suggest that this might also be the case in humans. The underlying mechanisms behind the reduction are yet to be determined, but it is tempting to speculate that the anti-inflammatory effects of the O-BN diet [30] are involved. In recent years, a number of preclinical studies have contributed to the development of a concept known as the microbiota–gut–brain axis [45]. The hypothesis proposes that age and unhealthy dietary components affect the gut mucosa and cause a gut leakage of proinflammatory agents (such as bacterial lipopolysaccharides (LPSs)) and primed immune cells. The agents trigger a low-grade chronic systemic inflammation, which in the long run affects the BBB and induces neuroinflammatory events in the brain. A direct link between gut microbiota and astrocytes (the so-called gut–astrocyte axis) has also been proposed, with a number of clinical and preclinical studies suggesting that astrocytic function is specifically modulated by the microbiome (for a review, see [46]). Our previous studies show that the O-BN diet profoundly alters the gut microbiota [47] and reduces the levels of circulating proinflammatory cytokines [48], which could, in turn, according to the microbiota–gut–brain and/or gut–astrocyte axis hypotheses, reduce neuroinflammatory events and thereby underly the reduction in plasma GFAP levels. The correlations between a number of cytokines and GFAP found in our current study are supportive of such an idea, but the GFAP levels did not correlate with gut leakage markers, and we have not been able to show an impact of the O-BN diet on gut leakage in our previous studies [30,33]. 

Inflammatory processes are also associated with T2D in a different aspect. In patients with obesity (which is strongly associated with T2D), the adipose tissue/cells become chronically inflamed, leading to increased levels of circulating proinflammatory cytokines. The cytokines have a detrimental impact, as they induce insulin resistance by inhibiting insulin signal transduction [49]. The systemic inflammation and elevated levels of circulating cytokines [50] may further provoke neuroinflammatory events in the brain. Notably, we found significant positive correlations between plasma GFAP levels and C-peptide at baseline, as well as insulin and insulin resistance, after 3 months in our study. Further support for this idea can be found in studies demonstrating activation of microglia and astrocytes after peripherally administered LPS injections, with increased cytokine secretion and upregulated GFAP as a result [51,52,53]. Indeed, increased GFAP expression has been detected in brains of different rodent diabetes models [27], although similar studies have not been performed on human brain samples yet. To our knowledge, only one published study has compared plasma GFAP levels between controls and patients with T2D. This study found elevated levels of GFAP in T2D, but the levels were not significantly different, and the comparison was not adjusted for age [19]. Nevertheless, the reported increase in GFAP autoantibodies in T2D patients points towards an alteration in GFAP expression in these patients. 

Importantly, although the levels of GFAP in blood (plasma or serum) have, in a number of studies, been used as an indicator of activation of astrocytes in the CNS [17], an increasing number of studies also show evidence for GFAP expression in some peripheral cells, including the enteric glia in the gastrointestinal tract. The enteric glia play a role in a wide range of gut functions, including maintaining the gut barrier function and, similar to astrocytes, supporting and communicating with neurons. These glia are also immunoreactive, and some of the enteric glial cell types become hypertrophic [54], proliferate [55,56], and upregulate a variety of proteins, including GFAP [57]. It may thus be that a proportion of the reduced GFAP in plasma seen after the O-BN diet reflects, at least partly, a reduction in reactive enteric glia. However, it should be pointed out that the majority of plasma GFAP is considered to be CNS-derived. In addition, since we found no correlations between the plasma GFAP and albumin or zonulin in plasma or feces or short-chain fatty acids in plasma at baseline or after the diet, we have no results supporting the link between the plasma GFAP levels and gut leakage. We did, however, find a correlation between GFAP and NfL, a plasma marker that is suggested to reflect neuronal alterations. This finding is in line with a previous study [58] and suggests a direct link between the plasma GFAP levels and brain alterations. Since our previous study has also shown improved psychological well-being after 3 months of the O-BN diet [30], we expected to find a correlation between the GFAP levels and well-being of the patients, but such a correlation was not found. Of note, the well-being was self-assessed using a validated VAS-scale 0–100, a method of low sensitivity [59], which may have influenced the possibility of detecting a potential link between the O-BN diet-reduced GFAP levels and improved psychological well-being.

The results found in this study should be interpreted with care, as there are some limitations to consider. First of all, the cohort size is rather small, and further studies on larger cohorts are encouraged. Secondly, the study lacks a rigorous clinical evaluation of psychological well-being, and specific memory and psychological health tests should be used in future studies. The strength of this study lies in the use of a pre–post design, where we assess the dietary intervention’s impact by comparing participant’s outcomes before and after the intervention, thereby effectively controlling for inter-individual variability.

## 5. Conclusions

To conclude, the observed reduction in plasma GFAP levels after following the O-BN diet for 3 months suggests that this diet may help mitigate factors that are strongly associated with neuroinflammation and cognitive decline. While it remains to be determined whether this effect involves the microbiota–gut–brain axis or other mechanisms of systemic inflammation, further research is warranted. Given the role of neuroinflammation in various neurodegenerative disorders associated with T2D, exploring the impact of diet on neuroinflammation is crucial to determine if specific dietary patterns can serve as preventive measures against the risk of developing these disorders. 

## Figures and Tables

**Table 1:** Plasma GFAP levels across different subgroups.

	Plasma GFAP Levels, pg/mL		
	Baseline	After 3 Months of Diet	After Unrestricted Eating
Sex			
Male	249.6 (200.8, 297.1)	229.2 (188.5, 266.5)	206.7 (180.4, 324.2)
Female	277.5 (226.3, 317.7)	287.5 (197.7, 325.0)	268.8 (227.8, 365.5)
*p*-value	*p* = 0.300	*p* = 0.133	*p* = 0.211
*APOE4* status			
Carrier	255.4 (242.1, 322.5)	272.9 (210.0, 328.3)	217.9 (180.4, 295.4)
Non-carrier	269.6 (206.7, 306.3)	243.8 (184.2, 302.5)	268.8 (205.1, 350.8)
*p*-value	*p* = 0.703	*p* = 0.471	*p* = 0.249
Compl. status			
At least one	277.5 (225.0, 322.5)	272.9 (229.2, 321.7)	315.0 (180.4, 359.6)
None	252.9 (208.3, 267.1)	210.0 (184.2, 277.1)	232.5 (204.2, 271.2)
*p*-value	*p* = 0.287	*p* = 0.047	*p* = 0.190

Data represent the medians (Q1, Q3) of plasma glial fibrillary acidic protein (GFAP) levels across different subgroups and were analyzed with the Mann–Whitney U Test. *APOE4*—Apolipoprotein E4, compl.—complication. The differences were considered significant when *p* ≤ 0.05.

**Table 2:** Correlations between plasma levels of GFAP and metabolic, peripheral inflammation, and CNS markers.

	Baseline	^a^ Baseline	^b^ Baseline
Age	0.482 **	-	-
IAPP	ns	0.500 **	0.478 *
C-peptide	0.375 *	ns	ns
HDL	ns	ns	−0.397 *
Triglycerides	0.418 *	ns	ns
IL1α	ns	0.453 *	0.440 *
IL4	ns	0.465 *	0.461 *
IL12p70	ns	0.500 **	0.490 **
IFNγ	ns	0.546 **	0.530 **
TNFα	ns	0.459 *	0.446 *
NfL	0.400 *	0.463 *	0.456 *

Data represent R-values of correlations between GFAP and other variables. ^a^ Adjusted for age. ^b^ Adjusted for age, sex, and body mass index (BMI). CNS—central nervous system, HDL—high-density lipoprotein, IAPP—islet amyloid polypeptide, IFN—interferon, IL—interleukin, NfL—neurofilament light polypeptide, TNF—tumor necrosis factor. Data were analyzed with Spearman and Partial Correlation tests, in the latter adjusting for age, ns—not significant. *—*p* ≤ 0.05, **—*p* ≤ 0.01.

## Data Availability

The data presented in this study are available on reasonable request from the corresponding author.
